# Quantitative high-throughput analysis of tumor infiltrating lymphocytes in breast cancer

**DOI:** 10.3389/fonc.2022.901591

**Published:** 2022-09-05

**Authors:** Kumiko Hayashi, Daichi Nogawa, Maki Kobayashi, Ayaka Asakawa, Yae Ohata, Shota Kitagawa, Kazuishi Kubota, Hisashi Takahashi, Miyuki Yamada, Goshi Oda, Tsuyoshi Nakagawa, Hiroyuki Uetake, Iichiroh Onishi, Yuko Kinowaki, Morito Kurata, Masanobu Kitagawa, Kouhei Yamamoto

**Affiliations:** ^1^ Department of Specialized Surgery, Graduate School of Medicine and Dentistry, Tokyo Medical and Dental University, Tokyo, Japan; ^2^ Department of Comprehensive Pathology, Graduate School of Medicine and Dentistry, Tokyo Medical and Dental University, Tokyo, Japan; ^3^ Molecular Pathology Group, Translational Research Department, Daiichisankyo RD Novare, Tokyo, Japan; ^4^ Department of Respiratory Medicine, Graduate School of Medicine and Dentistry, Tokyo Medical and Dental University, Tokyo, Japan; ^5^ Department of Medical Biochemistry and Microbiology, Science for Life Laboratory, Uppsala University, Uppsala, Sweden; ^6^ Department of Translational Science, Daiichi Sankyo, Inc., Basking Ridge, NJ, United States; ^7^ Department of Human Pathology, Graduate School of Medicine and Dentistry, Tokyo Medical and Dental University, Tokyo, Japan

**Keywords:** breast cancer, tumor microenvironment, tumor infiltrating lymphocytes, multiplex immunohistochemistry, PD1, PDL1, FOXP3

## Abstract

In breast cancer (BC), the development of cancer immunotherapy including immune checkpoint inhibitors has progressed. Tumor infiltrating lymphocytes (TILs) is one of the important factors for an immune response between tumor cells and immune cells in the tumor microenvironment, and the presence of TILs has been identified as predictors of response to chemotherapy. However, because complex mechanisms underlies the crosstalk between immune cells and cancer cells, the relationship between immune profiles in the tumor microenvironment and the efficacy of the immune checkpoint blocked has been unclear. Moreover, in many cases of breast cancer, the quantitative analysis of TILs and immuno-modification markers in a single tissue section are not studied. Therefore, we quantified detailed subsets of tumor infiltrating lymphocytes (TILs) from BC tissues and compared among BC subtypes. The TILs of BC tissues from 86 patients were classified using multiplex immunohistochemistry and an artificial intelligence-based analysis system based on T-cell subset markers, immunomodification markers, and the localization of TILs. The levels of CD4/PD1 and CD8/PD1 double-positive stromal TILs were significantly lower in the HER2- BC subtype (p <0.01 and p <0.05, respectively). In triple-negative breast cancer (TNBC), single marker-positive intratumoral TILs did not affect prognosis, however CD4/PDL1, CD8/PD1, and CD8/PDL1 double-positive TILs were significantly associated with TNBC recurrence (p<0.05, p<0.01, and p<0.001, respectively). TIL profiles differed among different BC subtypes, suggesting that the localization of TILs and their tumor-specific subsets influence the BC microenvironment.

## Introduction

Breast cancer (BC) is one of the most common cancers worldwide. Standard treatment options for BC include surgical resection, chemotherapy, hormone therapy, and HER2-targeted immunotherapy (1, 2). In addition to these conventional therapies, the development of cancer immunotherapy, including immune checkpoint inhibitors, is expected to improve patient survival (3–6). However, the mortality rate for BC remains high due to metastasis and recurrence. The development of new therapies and an understanding of tumor molecular and microenvironmental details is, therefore, important.

The tumor microenvironment (TME) is composed of various cell types such as lymphocytes, macrophages, fibroblasts, endothelial cells, and pericytes, with abundant extracellular matrix (7). The complex network of diverse cells and signaling pathways is closely related to tumor progression (7). The presence of lymphocytes in the tumor, referred to as tumor infiltrating lymphocytes (TILs) is evidence of a host immune response against the tumor cells (8). It has been reported that a high density of CD8-positive T cells in cancer nests is correlated with a favorable prognosis in various types of cancers (9–12). Moreover, the presence of TILs has been identified as a predictor of response to neoadjuvant chemotherapy in several human malignancies such as breast cancers (13, 14), hypopharyngeal cancers (15), and rectal cancers (8).

However, because complex mechanisms underlie the crosstalk between immune cells and cancer cells, the relationship between immune profiles of the TME is not fully understood. T lymphocytes are classified into CD4- and CD8-positive T cells based on their classical functional differences, which are further subcategorized based on various immunomodulatory functions (16). Regulatory T cells (Tregs) are a subset of T cells that negatively regulate immunity (17). Tregs express FOXP3, a transcription factor that plays an essential role in its differentiation, functional expression, and maintenance of differentiation state. FOXP3 is strongly and constantly expressed in Tregs among T cells and has been used as a marker for Tregs (17). FOXP3 increases the expression of CD25 and CTLA4 and reduces tumor immunity by suppressing the production of effector cytokines such as IL-2, IFNγ, IL-4, and IL-17. Infiltration of FOXP3-positive cells in various cancers has been reported to correlate with tumor stage and poor prognosis (18–20).

Immune checkpoints are known to regulate immune function *via* ligands and receptors (21). Regulators include the PD1-PDL1 system and the CD80-CTLA4 system, both of which negatively regulate tumor immunity by signaling tumor and stromal cell ligands to receptors on the T-cell surface (22). Immune checkpoint therapy, which activates tumor immunity by inhibiting this system, has recently been focused on as a therapeutic strategy for refractory cancers (23). TILs are classified into stromal TILs (sTILs), which invade the stroma near the tumor, and intratumoral TILs (iTILs), which invade the tumor itself (24). The clinical and biological significance of sTILs and iTILs have been studied, and various significant differences have been reported for each TIL subtype (25, 26). While TILs have complex temporal and spatial effects due to their various functions and localization, their significance is not yet clear, owing to technical limitations in previous studies. To overcome these limitations, it is necessary to evaluate TIL profiles by using high-quality multiple staining on the same section, objective measurement of complex markers, and localization of TILs.

In this study, we used state-of-the-art methods to quantify a detailed subset of TILs and to understand how they differ among BC subtypes. Furthermore, the significance of TILs in the clinical outcomes of triple-negative breast cancer (TNBC) was examined.

## Materials and methods

### Samples

Formalin-fixed paraffin-embedded (FFPE) samples (n = 86) of breast invasive ductal carcinoma obtained from the Tokyo Medical and Dental University Hospital, Tokyo, between 2014 and 2017, were used in this study. The specimens were obtained by surgical resection, routinely fixed in 10% neutralized formalin, and then embedded in paraffin for conventional histopathological examination. For immunohistochemistry and multiplex immunohistochemistry, FFPE tissue (4 μm in thickness) was sliced, and the sections were placed on silane-coated slides. This study was approved by the ethics committees of Tokyo Medical and Dental University (M2018-141) and Daiichi Sankyo RD Novare Co. Ltd. (N18-0082-00), and all procedures were performed in accordance with the ethical standards established by these committees.

### Immunohistochemistry for ER, PgR, HER2, and case categorization

To classify BC samples into each subtype, immunostaining for estrogen receptor (ER), progesterone receptor (PR), and HER2/neu (HER2) was performed. For ER and PR, the commercially available immunostaining kit (® Histofine ER/PgR (MONO)) and universal kit (Nichirei Biosciences Japan, Tokyo, Japan)) were used. HER2 IHC staining was performed with VENTANA anti-HER2/neu (4B5) Rabbit Monoclonal Primary Antibody [VENTANA pathway HER2 (clone: 4B5)] (Roche Diagnostics, Japan) and ultra VIEW DAB Detection Kits (Roche Diagnostics) using an automated slide stainer, Ventana BenchMark ULTRA (Roche Diagnostics). The sections were incubated with primary antibody [PATHWAY HER2 (4B5), Roche diagnostics] for 16 min at 36°C. Expression levels of HER2 in tumor cells were defined as 0 (absent), 1 (weak, incomplete membrane staining), 2 (weak to moderate, complete membrane staining), and 3 (strong and complete membrane staining). Based on the American Society of Clinical Oncology (ASCO)/College of American Pathologists (CAP) guidelines, HER2 status was classified into 4 groups, IHC 3+ (intensity 3 staining observed in > 10% of tumor cells), IHC 2+ (intensity 2 staining in >10% of tumor cells), IHC 1+ (intensity 1 staining in ≤10% of tumor cells), and IHC 0 (no staining was observed). If HER2 status was 3+, these cases were defined as HER2-positive BC (HER2). When HER2 status was 2+, HER2 fluorescence *in situ* hybridization was performed by an external company (SRL Tokyo, Japan). If the HER2 signal was more than 1.7, these cases were also defined as HER2. When the HER2 status was 0, 1+, and 2, ER or PgR-positive cases were defined as ER/PgR-positive BC (ER/PR), and ER or PgR-negative cases were defined as TNBC. These determinations were made by two pathologists (KY and MORITO KURATA(MK)).

### Multiplex immunohistochemistry

In this study, mIHC staining was performed using the Opal 7-color Automation IHC kit (Akoya Bioscences, MA, USA) with an automated slide stainer, BOND RX (Leica, Wetzlar, Germany). The primary antibodies used in this study were as follows: PD1(1:200, NAT105, Abcam, Cambridge, UK), CD8 (1:350, C8/144B, Agilent, Santa Clara, CA, USA), CD4 (1:50, 4B12, Leica), FOXP3 (1:300, D608R, Cell Signaling Technologies, Beverly, MA, USA), PDL1 (1:100, SP142, Abcam), and pan-cytokeratin (Pan-CK) (1:4, AE1/AE3, Agilent). The staining protocol was performed in accordance with the manufacturer’s instructions by using the Opal 7-color automation IHC kit and BOND research detection kit. Each primary antibody was incubated for 30 min at room temperature. Slides were mounted with ProLong Diamond Antifade Mountant (Thermo Fisher Scientific, Waltham, MA, USA).

### Quantitative image analysis

**Figure 1:**
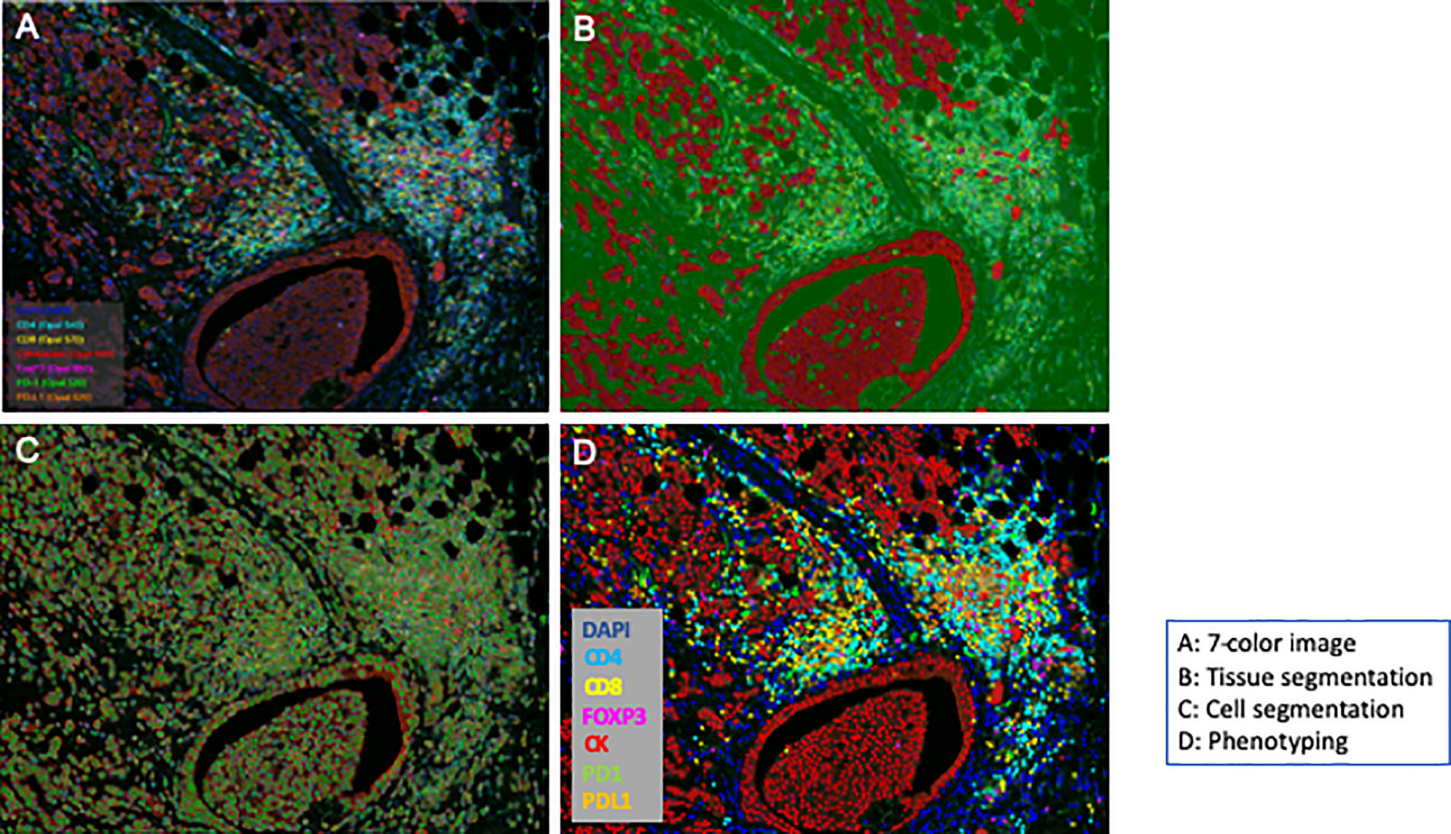
Workflow of an automated multispectral imaging system. Fluorescence multiplexed immunohistochemistry and merged images. Whole section was segmented based on the expression of cytokeratin (segmentation); each image was captured (cell detection) and unmixed spectrally to quantify each marker (phenotyping).

The 86 stained slides were subjected to 7-color multispectral image analysis using an automated quantitative pathology imaging system, Vectra Polaris (Akoya Bioscences, MA, USA). Each whole slide imaging was scanned at 10× magnification in order to select high-powered multispectral imaging at 20× (resolution of 0.5 μm per pixel, 931 μm × 698 μm) by using Phenochart viewer (version 1.0, Akoya Bioscences, MA, USA). One sample was excluded because there were almost no cancer cells in the specimen. For quantitative imaging, five areas per slide were randomly selected as each sTIL area and iTIL area as regions of interest (ROIs) for each section according to a method proposed by the International TILs Working Group 2014 (27). None of the sections contained normal epithelial cells. After scanning the slides, image files generated by Vectra Polaris were analyzed using the image analysis software inForm (version 2.4.0., Akoya Bioscences). Specifically, 7-color image preparation, trainable tissue segmentation using an AI-based algorithm, adaptive cell segmentation, and scoring for positive cell percentage of tumor and stroma area were performed (**Figures 1A–D**). Pan-CK staining results were used to recognize tumoral areas during segmentation and for AI training. After the above trainable methods, the scoring method counted the positive cells in each ROI (0.246 mm^2^), which were corrected per 1 mm^2^ above a specific threshold of each marker. Single positive scoring (CD4, CD8, FOXP3, PD1, PDL1) of each marker and double positive scoring (CD4-PD1, CD4-PDL1, CD4-FOXP3, CD8-PD1, CD8-PDL1, CD8-FOXP3) were calculated.

### Relapse-free survival analysis

The recurrence period after surgery for TN subtype 22 cases were investigated and classified into “high” and “low” groups by the amount of each TIL. The two groups were classified based on the median. These were then used to compare the prognosis of each recurrence-free survival by using the Kaplan-Meier method.

### Data analysis and statistical procedures

All scoring data generated by inForm were collected and analyzed. The status of the number of positive cells per 1 mm^2^ for TIL markers in each cancer subtype (ER/PR, HER2, TN) were compared. One-way ANOVA and Tukey’s multiple comparison tests were used to determine statistically significant differences in the unpaired data for scoring of each marker. Statistical analyses were performed using GraphPad Prism version 9.2 (GraphPad Software), and statistical significance was set at p < 0.05.

## Results

### Patient characteristics

The characteristics of the cases used in this study are summarized in **Table 1**. A total of 86 breast invasive ductal carcinoma (BIDC) patients with T stage I (46 patients), T stage II (30 patients), T stage III (8 patients), and T stage IV (2 patients) were included in this study. The study group consisted of women with a median age of 61 years (range, 32–88 years). Lymph node metastasis was positive in 25 cases and negative in 50 cases. Immunostaining revealed the ER/PR subtype in 46 cases (53%), HER2 subtype in 18 cases (21%), and TNBC subtype in 22 cases (26%). Multi-colored immunohistochemical analyses were performed on these cases as described in the materials and methods section and discussed in the results below.

**Table 1:** Clinicopathological features of patients included in this study.

Clinicopathological feature		ER/PR (n=46)	HER2 (n=18)	TN (n=22)
Age	Median (range)	58 (32-86)	52 (33-71)	63 (36-88)
pT classification	T1	26	11	9
	T2	15	7	8
	T3	4	0	4
	T4	1	0	1
Neoadjuvant chemotherapy	+	6	2	8
	–	40	16	14
Lymph node metastasis	Positive	15	6	4
	Negative	29	9	12
	NX	2	3	6

### Representative histology of multi-colored immunostaining

**Figure 2:**
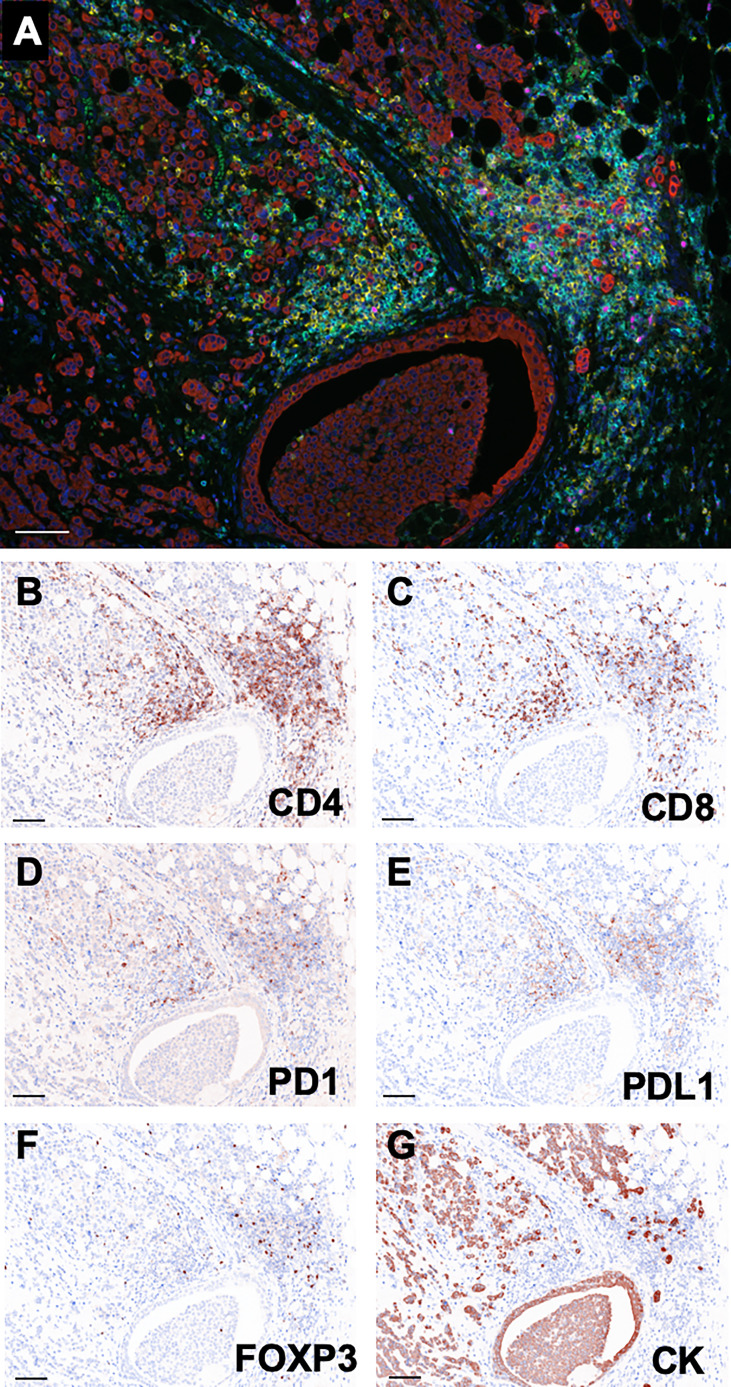
Representative multi-colored immunostaining and virtual pathological images. **(A)** Composite image (7-color image) of representative lesion. Virtual pathological image of **(B)** CD4, **(C)** CD8, **(D)** PD1, **(E)** PDL1, **(F)** FOXP3, **(G)** Pan-CK in a single tissue section. Brownish-colored areas indicate a positive signal. Scale bars indicate 100μm.

As shown in **Figure 2A**, single tissue sections were successfully stained with PD1 (green), CD8 (yellow), CD4 (blue), PDL1 (orange), FOXP3 (pink), Pan-CK (red). The virtual pathological image of each staining was performed to confirm that each immunostaining was reasonably functional (**Figures 2B–G**).

### Quantitative comparison of sTILs among BC subtypes

The amounts of sTILs in the three subtypes are shown in **Supplementary Figures 1A–C**. In each group, CD4+ sTILs were the most common, followed by CD8+ sTILs (ER/PR; 2418.7± 4233.2, HER2; 3374.2 ± 4906.2, TN; 3345.6 ± 5873.6). Next, the quantitative differences between the subtypes of each sTILs were examined. As shown in **Supplementary Figures 2A–E** and, the number of CD8+ sTILs in the ER/PR subtype were significantly lower than that in HER2 (p<0.001) and TN subtypes (p=0.024) (**Supplementary Figure 2B**). The number of PD1+ sTILs in the HER2 subtype was significantly lower than that in the ER/PR (p=0.017) and TN (p=0.003) subtypes (**Supplementary Figure 2C**). The number of PDL1+ sTILs in the ER/PR subtype were significantly lower than that in the HER2 (p<0.001) and TN (p<0.001) subtypes (**Supplementary Figure 2D**), and the number of FOXP3+ sTILs in the HER2 subtype were significantly higher than that in the ER/PR (p<0.001) and TN (p=0.001) subtypes (**Supplementary Figure 2E**). The proportions of cells with each sTIL among the subtypes are shown in **Supplementary Table 1**.

**Figure 3:**
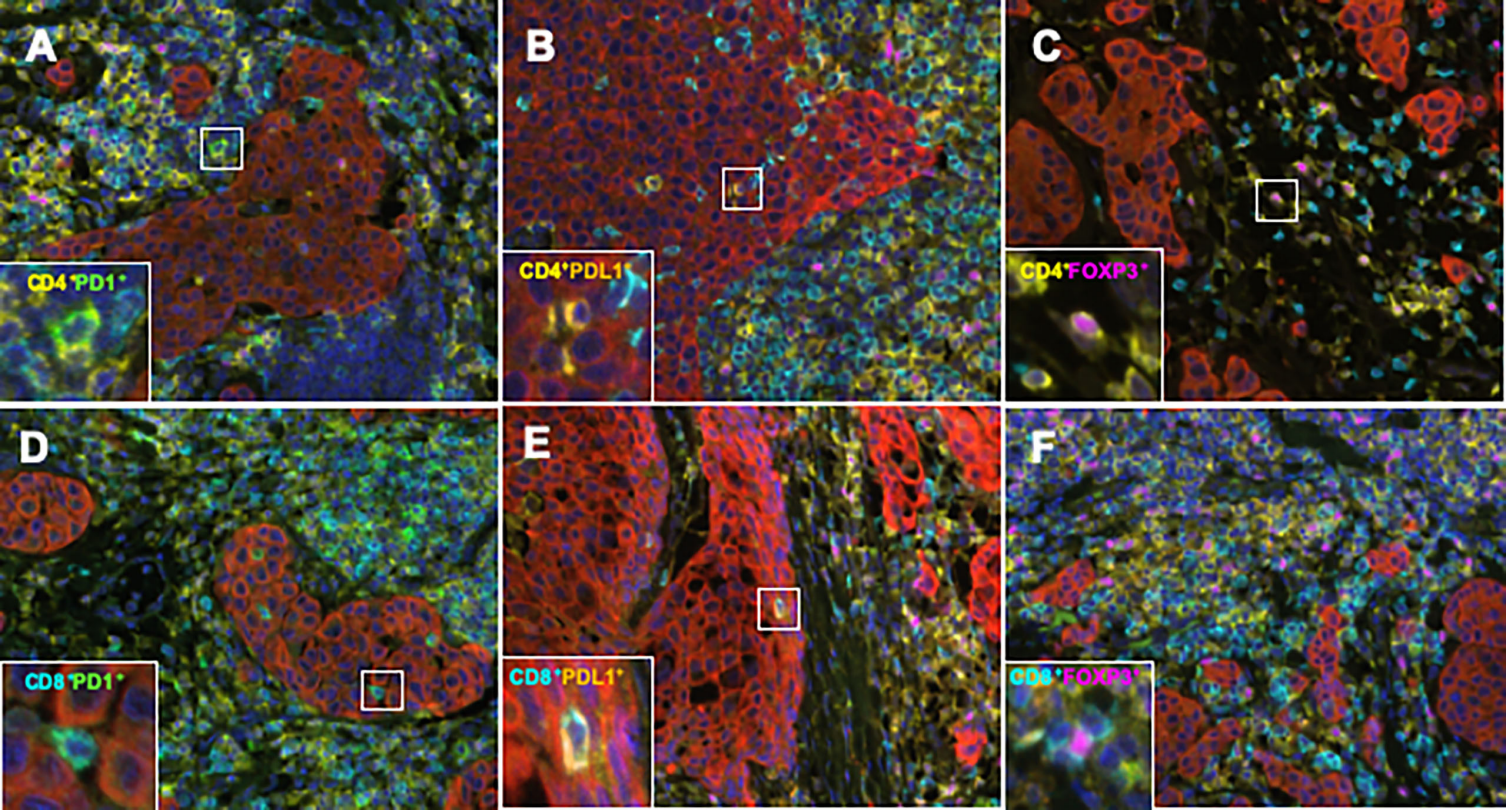
Representative TIL marker double-positive cells obtained using an automated quantitative pathology imaging system and image analysis software. **(A)** CD4 (yellow)/PD1 (green), **(B)** CD4 (yellow)/PDL1 (orange), **(C)** CD4 (yellow)/FOXP3 (pink), **(D)** CD8 (light blue)/PD1 (green), **(E)** CD8 (light blue)/PDL1 (orange), and **(F)** CD8 (light blue)/FOXP3 (pink) double positive cells in BC tissue.

**Figure 4:**
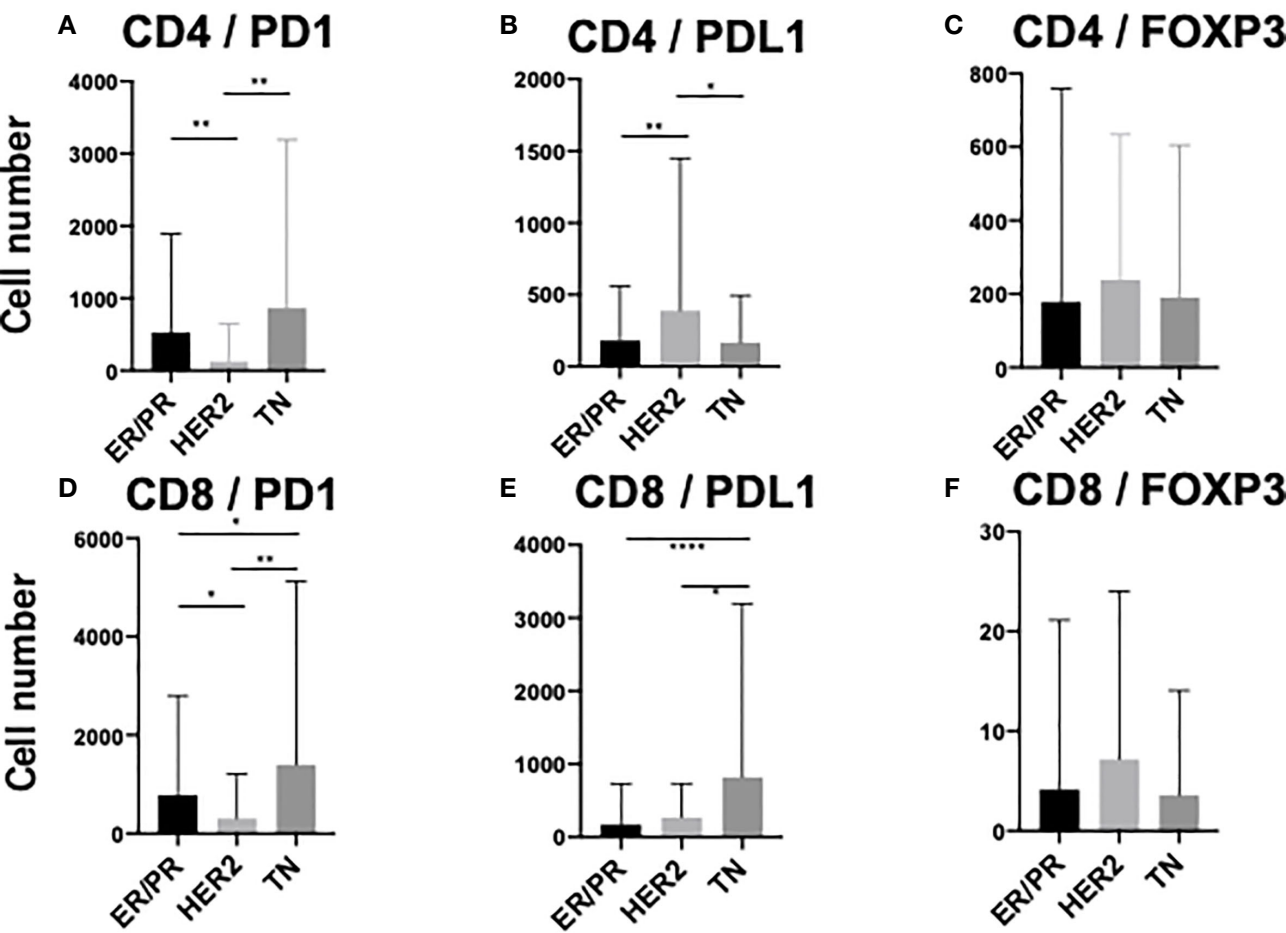
Comparison of stromal TIL (sTIL) marker double-positive cells among ER/PR, HER2, and TN subtypes. Average number of **(A)** CD4/PD1, **(B)** CD4/PDL1, **(C)** CD4/FOXP3, **(D)** CD8/PD1, **(E)** CD8/PDL1, and **(F)** CD8/FOXP3 double-positive sTILs among each subtype (ER/PR: n=46, HER2: n=18, TN: n=22). *, **, and **** indicate p < 0.05, p < 0.01, and, p< 0.0001 respectively (One way ANOVA test and Tukey’s multiple comparison test). Error bars represent standard deviation.

To elucidate the detailed subpopulation of sTILs, double-positive sTILs were analyzed. Images for double-positive visualization are shown in **Figures 3A–F**. **Supplementary Figure 3** shows the number of double-positive sTILs in each of the three subtypes. CD8/PD1-double positive sTIL levels were higher in the ER/PR and TN subtypes (ER/PR; 783.2 ± 2017.1, TN; 1399.6 ± 3706.2). CD4/PDL1 positive sTIL levels were the highest in the HER2 subtype (389.4 ± 1056.2). Among the three subtypes (**Figures 4A–F**), CD4/PD1-double positive sTIL levels were significantly lower in HER2 than in the ER/PR (p=0.004) and TN subtypes (p=0.002) (**Figure 4A**). The number of CD4/PDL1-double positive sTILs in the HER2 subtype were significantly higher than that in the ER/PR (p=0.008) and TN (p=0.03) subtypes (**Figure 4B**). The number of CD8/PD1-double positive sTILs in HER2 were significantly lower than that in the ER/PR (p=0.025) and TN (p=0.005) subtypes (**Figure 4D**). The number of CD8/PDL1-double positive sTILs in the TN subtype were significantly higher than that in both the ER/PR (p<0.001) and HER2 (p=0.023) subtypes (**Figure 4E**). The proportions of cells with each sTIL among the subtypes are shown in **Supplementary Table 2**.

### Quantitative comparison of iTILs among BC subtypes

The amounts of iTILs in the three subtypes are shown in **Supplementary Figures 4A–C**. Unlike sTILs, PD1 positive iTIL levels were the highest in the ER/PR and TN subtypes (ER/PR: 2242.5 ± 7657.8, TN: 1826.8 ± 11564.4), while FOXP3-positive iTIL levels were highest in the HER2 subtype (365.9 ± 647.7). Next, the quantitative differences between the iTIL subtypes were examined. As shown in **Supplementary Figures 5A–E**, the number of CD4+ iTILs in the TN subtype was significantly higher than that in the ER/PR subtype (p<0.005) (**Supplementary Figure 5A**). The number of CD8+ iTILs in the TN subtype was significantly higher than that in the ER/PR subtype (p=0.01) (**Supplementary Figure 5B**), and there were significantly more FOXP3+ iTILs in the TN subtype than in the ER/PR (p=0.036) and HER2 subtypes (p=0.028) (**Supplementary Figure 5E**). The proportions of cells with each iTIL among the subtypes are shown in **Supplementary Table 3**.

**Figure 5:**
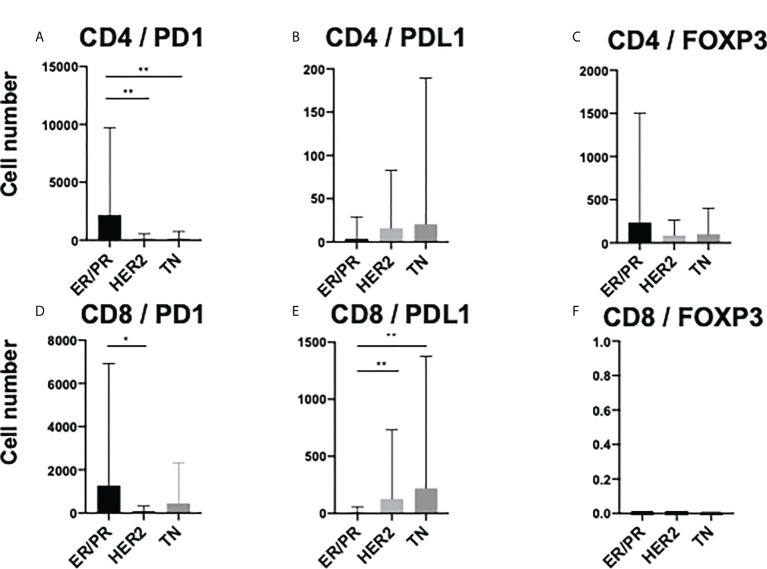
Comparison of TIL marker double-positive cells amongst intratumoral TILs (iTILs) in ER/PR, HER2, and TN subtypes. Average number of **(A)** CD4/PD1, **(B)** CD4/PDL1, **(C)** CD4/FOXP3, **(D)** CD8/PD1, **(E)** CD8/PDL1, and **(F)** CD8/FOXP3 double-positive iTILs among each subtype (ER/PR: n=46, HER2: n=18, TN: n=22). *, **, and indicate p < 0.05, p < 0.01, respectively (One-way ANOVA and Tukey’s multiple comparison test). Error bars represent standard deviation.

To elucidate the detailed subpopulation of iTILs, double-positive iTILs were analyzed. **Supplementary Figure 6** shows the number of double-positive iTILs in each of the three subtypes. CD4/PD1-double positive iTIL levels were higher in the ER/PR and HER2 subtypes (ER/PR, 2164.5± 7549.8; HER2, 160.1± 384.7). CD8/PD1 positive iTIL levels were the highest in the TN subtype (444.7 ± 1880.1). Among the three subtypes (**Figures 5A–F**), CD4/PD1-double positive iTIL levels were significantly higher in the ER/PR subtype than in the HER2 (p=0.009) and TN subtypes (p=0.004) (**Figure 5A**). The number of CD8/PD1-double positive iTILs were significantly higher in the ER/PR subtype than in the HER2 subtype (p=0.035) (**Figure 5D**). The number of CD8/PDL1-double positive iTILs were significantly lower in the ER/PR subtype than in the HER2 (p=0.003) and TN (p=0.006) subtypes (**Figure 5E**). The proportions of cells with each iTIL among the subtypes are shown in **Supplementary Table 4**.

### Relationship between TIL and recurrence in the TN subtype

Of the three subtypes, the relationship between the number of TILs and recurrence was investigated in the TN subtype, which has a relatively higher rate of recurrence and metastasis (28).

**Figure 6:**
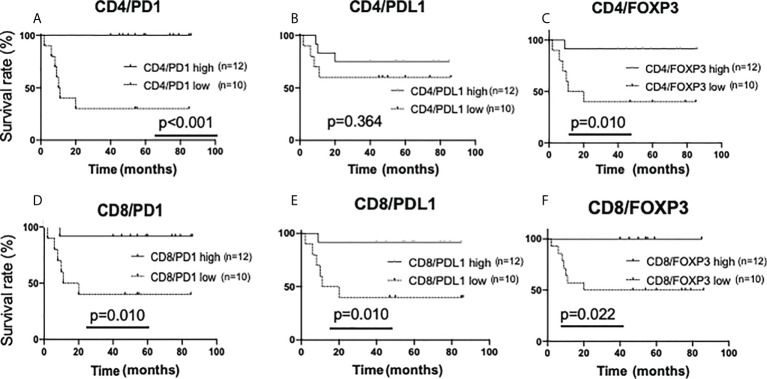
Kaplan-Meier analysis for recurrence-free survival categorized by the amount of stromal TIL (sTIL) marker double-positive cells in triple-negative cases. In TN subtypes (n=22), each sTIL marker was classified into “low” (n=10) and “high” (n=12)and analyzed as follows. **(A)** CD4/PD1-double positive sTIL low; <80 cells, high; >=80 cells, **(B)** CD4/PDL1-double positive sTIL low; <50 cells, high; >=50 cells, **(C)** CD4/FOXP3-double positive sTIL low; <60 cells, high; >=60 cells, **(D)** CD8/PD1-double positive sTIL low; <120 cells, high; >=120 cells, **(E)** CD8/PDL1-double positive sTIL low; <85 cells, high; >=85 cells, **(F)** CD8/FOXP3-double positive sTIL low; =<0 cells, high; >0 cells.

First, the amount of sTILs and recurrence-free survival rates were investigated (**Supplementary Figures 7A–E**). For the single staining analysis in all sTILs, the “low” groups had a higher rate of recurrence than the “high” groups (CD4; p=0.007, CD8; p<0.001, FOXP3; p=0.094, PD1; p=0.006, PDL1; p=0.074). In double staining analysis, in all sTILs except CD4/PDL1, the “low” groups had a statistically higher rate of recurrence than the “high” groups (**Figures 6A–F**).

**Figure 7:**
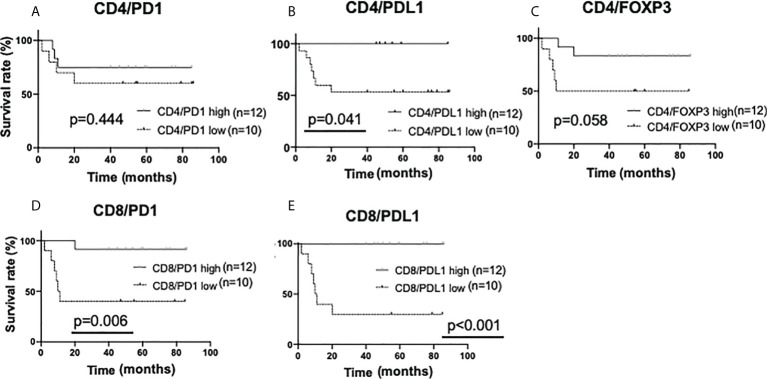
Kaplan-Meier analysis for recurrence-free survival categorized by the amount of intratumoral TIL (iTIL) marker double-positive cells in triple-negative cases. In TN subtypes (n=22), each sTIL marker was classified into “low” (n=10) and “high”(n=12) and analyzed as follows. **(A)** CD4/PD1-double positive iTIL low; <15 cells, high; >=15 cells, **(B)** CD4/PDL1-double positive iTIL low; =<0 cells, high; >0 cells, **(C)** CD4/FOXP3-double positive iTIL low; <30 cells, high; >=30 cells, **(D)** CD8/PD1-double positive iTIL low; <30 cells, high; >=30 cells, **(E)** CD8/PDL1-double positive iTIL low; <6 cells, high; >=6 cells.

Next, the amounts of sTILs and iTILs were each stratified and examined. In the analysis by single staining (iTILs), no significant difference in recurrence tendency was observed due to the difference in the amount of iTILs (**Supplementary Figures 8A–E**). Interestingly, in the double staining iTIL analysis, a low number of CD4/PDL1, CD8/PD1, and CD8/PDL1-double positive iTILs had a higher rate of recurrence than the ones with higher numbers (CD4/PDL1, p=0.041; CD8/PD1, p=0.006; CD8/PDL1, p<0.001) (**Figures 7A–E**).

## Discussion

In investigating the biological properties of tumors, it is very important to understand the environmental factors surrounding the tumor in addition to the properties of the tumor cells themselves (mRNA and protein expression, metabolism, and genome alteration). In addition to various humoral factors such as oxygen status, nutritional status, pH, cytokines, and hormones around tumor cells, cell elements such as TILs are involved in crosstalk with tumor cells to create what is referred to as the TME (29). Since TILs dynamically control both humoral and cellular responses, detailed profiling, including functional analysis of TILs, has provided insights for understanding the role of TME in various cancers (30–34).

In this study, we quantified sTILs and iTILs in different histological subtypes of BC by using mIHC and AI-equipped high-throughput immuhistochemical analysis (HTIA). There are many reports on the correlation between a TIL-rich environment and better clinical outcomes and prognosis in BC. Presence of TILs is an independent predictor of response to neoadjuvant therapy (9) and a good prognostic factor in TN (35, 36) and molecular-targeted therapy in the HER2 subtype (14, 37). Regarding the relationship between the quantitative viewpoint of TILs and prognostic factors, the data obtained in this study are limited to recurrence of TN; however, the results are consistent with those previously reported (36). Regarding the number of TILs determined for each subtype, it has been reported that the number of sTILs and iTILs tend to be higher in HER2 and TN subtypes than in ER/ER subtypes, with the numbers in TN subtypes being the highest (38). In this single-factor analysis after multiple staining, HER2 or TN subtypes had more TILs than the ER/PR subtypes for CD8, PDL1, and FOXP3, and the technical aspect of multiple staining was also regarded as acceptable.

There are two important points in advancing the detailed analysis of TILs. The first is an evaluation using different parts of the sTILs and iTILs, and the other is an evaluation using multiple markers by mIHC. The former contributes to the spatial assessment within the main tumor tissue, and the latter adds depth to the biological functions of TILs. In iTILs, a detailed definition of TILs was presented at the International TILs Working Group in 2014 (27). Furthermore, sTILs have better measurement reproducibility than iTILs, and unlike iTILs, they are not affected by tumor cell density or growth pattern, therefore, the evaluation of sTILs was considered better (27). In fact, there is a report suggesting discrepancy between observers regarding iTILs in BC (39). However, recent studies have reported that the CD4/CD8 ratio of sTILs is a poor prognostic marker of TN (40), and that both sTILs and iTILs are factors that predict the pathological response to Neoadjubant chemotherapy (NAC) (41). This suggests that both sTILs and iTILs are involved in shaping the TME and determining clinical outcomes. In this study, single marker analysis correlated sTILs depletion with a poor prognosis and increased recurrence probability. Comparisons with multiple marker analyses, as described below, could provide further insights into the role of iTILs in prognosis.

With the development of immunostaining in recent years, mIHC using fluorescent dyes has been used for pathological research, including TME analysis (33, 42–44). In lung cancer, it was reported that RNA sequences in TILs correlate with protein expression, as determined by mIHC, providing evidence for the clinical utility of mIHC (31). Furthermore, mIHC is also a useful tool to elucidate the mechanism of TME formation (45). Analysis of TILs by mIHC has also been performed in BC (46); however, the types of antibodies are limited. In this study, mIHC was performed with T-cell markers (CD4, CD8) and immune modification markers (PD1, PDL1, and FOXP3). Analysis of double-positive cells was performed using the AI-equipped HTIA system. We were able to perform objective, detailed, and functional TIL analysis through this methodology. It was shown that the decrease in CD4/FOXP3-double positive TILs, which are known to exist in the intratumoral region, and CD8/PD1-double positive TILs, which have most recently been reported (47), correlates with recurrence. It has been reported that CD8/PD1-double positive cells express T-cell exhaustion markers, such as TIM3, in the TME of primary brain lymphoma, resulting in an attenuated function in tumor immunity (34). Furthermore, it has been reported that invasive BCs do not have many CD8-positive sTILs but do have many CD8-positive iTILs which are a negative prognostic marker (26). Moreover, a recent study reported that CD103-positive iTILs are a favorable prognostic factor for TN subtypes (48). In this study, although the difference was not statistically significant, cases with high CD8-positive iTILs and low FOXP3-positive iTILs showed a tendency to recur, it is possible that CD8-positive iTILs are classified into positive and negative subtypes for tumor immune function, which can be clarified by prospective analysis using mIHC together with additional markers. Although these reports are contrary to the results of this study, it is possible that CD8-positive iTILs are classified into positive and negative subtypes for tumor immune function, which can be clarified by prospective analysis using mIHC together with additional markers.

CD4/PDL1-double positive TILs and CD8/PDL1-double positives have not been reported as subsets of lymphocytes. Although the numbers of these subsets are considerably lower than that of CD4/PD1-positive or CD8/PD1-positive TILs, they may be novel subsets specific to the TME. A detailed subset analysis using mIHC and flow cytometry with other markers in different cancer types is expected to provide more insights into the roles of these TILs in the TME.

This study has some limitations. First, TILs from five random areas per section were examined for every case; however, it is not clear whether these results reflect the entire TME. Recent developments in image analysis technology have made it possible to analyze TILs from the whole section (49). It is expected that a more accurate TME evaluation will be possible using this technology. In addition, macrophages in the TME—the tumor-associated macrophages (TAMs)—are also known to play important roles in tumor progression (50). Indeed, it has been revealed that TAMs express various effector molecules that inhibit the antitumor immune response (50–52). Multiplex immunohistochemical analysis for both TILs and TAMs will resolve the complexity of the TME in BC. In addition, eight from 22 cases of the TN cases were treated with NAC, and it is possible that NAC affected the TME. It would be very important to analyze the changes in TIL caused by NAC, as it may lead to the prediction of the therapeutic effect of NAC. Further investigation of the correlation between TIL changes before and after NAC, and treatment response is expected. Finally, in recent years, immune checkpoint inhibitors (ICIs) have become widely used as therapeutic molecules in BC, including the TN subtype. It has been reported that there is a close relationship between the therapeutic effect of ICI and TILs in various cancer types (42, 53). It is supposed that the techniques used in this study can be applied to advanced research areas. For example, by examining how the amount and distribution of various lymphocyte subsets change before and after administration of an immune checkpoint inhibitor (ICI), we can determine the histological characteristics and distribution of lymphocytes that directly reflect the effect of the drug. If this becomes clear, it may be possible to predict the efficacy of ICIs not only from tumor cell characteristics, but also from TME. It is expected that the findings from this study will provide basic insights into the TME in BC. Further studies will focus on elucidating the significance of TILs into a more detailed subset based on their classification, and its effect on therapies such as ICI and NAC to resolve unknown functions of TME.

## Data availability statement

The original contributions presented in the study are included in the article/**Supplementary Material**. Further inquiries can be directed to the corresponding author.

## Ethics statement

Ethical approval was granted by both of the ethics committees, Tokyo Medical and Dental University (M2018-141) and Daiichi Sankyo RD Novare Co. Ltd. (N18-0082-00). In accordance with the approval, informed consent was not required due to this being a historical surgical specimens. The study design was advertised in selected printed media prior to effectuation, with the possibility to opt-out, as specified in the approval.

## Author contributions

KH, DN, MaK, AA, and KY conceived the structure of the manuscript; KH and KY retrieved the relevant paper and finished the manuscript including figures and tables; YO, SK, KK, HT, and MY prepared pathology slides and performed experiments. GO, TN, and HU revised the manuscript. IO, YK, MoK, and KY diagnosed the subtypes of the 86 BC patients. All authors have contributed to the preparation of this manuscript. All authors read and approved the final manuscript.

## Funding

The authors declare that this study received funding from Daiichi Sankyo RD novare company. The funder was not involved in the study design, collection, analysis, interpretation of data, the writing of this article or the decision to submit it for publication.

## Acknowledgments

We would like to thank Editage (www.editage.jp) for English-language editing.

## Conflict of interest

Author KK was employed by Daiichi Sankyo, Inc.

The remaining authors declare that the research was conducted in the absence of any commercial or financial relationships that could be construed as a potential conflict of interest.

## Publisher’s note

All claims expressed in this article are solely those of the authors and do not necessarily represent those of their affiliated organizations, or those of the publisher, the editors and the reviewers. Any product that may be evaluated in this article, or claim that may be made by its manufacturer, is not guaranteed or endorsed by the publisher.

## References

[1] Lakhani SR Ellis IO Schnitt SJ Tan PH van de Vijver MJ. WHO classification of tumours of the breast, 4th ed. (2012). p13–32.

[2] Waks AG Winer EP. Breast cancer treatment: a review. JAMA (2019) 321:288–300. doi: 10.1001/jama.2018.19323 30667505

[3] Postow MA Callahan MK Wolchok JD. Immune checkpoint blockade in cancer therapy. J Clin Oncol (2015) 33:1974–82. doi: 10.1200/JCO.2014.59.4358 PMC4980573 25605845

[4] Pardoll DM. The blockade of immune checkpoints in cancer immunotherapy. Nat Rev Cancer (2012) 12:252–64. doi: 10.1038/nrc3239 PMC4856023 22437870

[5] Khalil DN Smith EL Brentjens RJ Wolchok JD. The future of cancer treatment: immunomodulation, CARs and combination immunotherapy. Nat Rev Clin Oncol (2016) 13:273–90. doi: 10.1038/nrclinonc.2016.25 PMC5551685 26977780

[6] Okazaki T Chikuma S Iwai Y Fagarasan S Honjo T. A rheostat for immune responses: the unique properties of PD-1 and their advantages for clinical application. Nat Immunol (2013) 14:1212–8. doi: 10.1038/ni.2762 24240160

[7] Whiteside TL. The tumor microenvironment and its role in promoting tumor growth. Oncogene (2008) 27:5904–12. doi: 10.1038/onc.2008.271 PMC3689267 18836471

[8] Kim R Emi M Tanabe K. Cancer immunoediting from immune surveillance to immune escape. Immunology (2007) 121:1–14. doi: 10.1111/j.1365-2567.2007.02587.x 17386080 PMC2265921

[9] Denkert C Loibl S Noske A Roller M Müller BM Budczies MK. Tumor-associated lymphocytes as an independent predictor of response to neoadjuvant chemotherapy in breast cancer. J Clin Oncol (2010) 28:105–13. doi: 10.1200/JCO.2009.23.7370 19917869

[10] Katz SC Bamboat ZM Maker AV Shia J Pillarisetty VG Yopp AC. Regulatory T cell infiltration predicts outcome following resection of colorectal cancer liver metastases. Ann Surg Oncol (2013) 20:946–55. doi: 10.1245/s10434-012-2668-9 PMC3740360 23010736

[11] Fridman WH Pagès F Sautès-Fridman C Galon J. The immune contexture in human tumours: impact on clinical outcome. Nat Rev Cancer (2012) 12:298–306. doi: 10.1038/nrc3245 22419253

[12] Mahmoud SM Paish EC Powe DG Macmillan RD Grainge MJ Andrew HS. Tumor-infiltrating CD8+ lymphocytes predict clinical outcome in breast cancer. J Clin Oncol (2011) 29:1949–55. doi: 10.1200/JCO.2010.30.5037 21483002

[13] Ono T Azuma K Kawahara A Sasada T Hattori S Sato F.. Association between PD-L1 expression combined with tumor-infiltrating lymphocytes and the prognosis of patients with advanced hypopharyngeal squamous cell carcinoma. Oncotarget (2017) 8:92699–714. doi: 10.18632/oncotarget.21564 PMC5696215 29190949

[14] Hou Y Nitta H Wei L Banks PM Parwani AV Li Z. Evaluation of immune reaction and PD-L1 expression using multiplex immunohistochemistry in HER2-positive breast cancer: the association with response to anti-HER2 neoadjuvant therapy. Clin Breast Cancer (2018) 18:e237–44. doi: 10.1016/j.clbc.2017.11.001 PMC7219558 29198959

[15] Park IJ An S Kim SY Lim HM Hong SM Kim MJ. Prediction of radio-responsiveness with immune-profiling in patients with rectal cancer. Oncotarget (2017) 8:79793–802. doi: 10.18632/oncotarget.19558 PMC5668093 29108360

[16] Zeng Z Chew HY Cruz JG Leggatt GR Wells JW. Investigating T cell immunity in cancer: achievements and prospects. Int J Mol Sci (2021) 22:2907. doi: 10.3390/ijms22062907 33809369 PMC7999898

[17] Hori S Nomura T Sakaguchi S. Control of regulatory T cell development by the transcription factor Foxp3. Science (2003) 299:1057–61. doi: 10.1126/science.1079490 12522256

[18] Li C Wang H Fang H He C Pei Y Gai X. FOXP3 facilitates the invasion and metastasis of non-small cell lung cancer cells through regulating VEGF, EMT and the notch 1/Hes 1 pathway. Exp Ther Med (2021) 22:958. doi: 10.3892/etm.2021.10390 34335900 PMC8290412

[19] Satoh K Kobayashi Y Fujimaki K Hayashi S Ishida S Sugiyama D.. Novel anti-GARP antibody DS-1055a augments anti-tumor immunity by depleting highly suppressive GARP+ regulatory T cells. Int Immunol (2021) 33:435–46. doi: 10.1093/intimm/dxab027 34235533

[20] Ghods A Mehdipour F Shariat M Talei AR Ghaderi A. Regulatory T cells express tumor necrosis factor receptor 2 with the highest intensity among CD4+ T cells in the draining lymph nodes of breast cancer. Mol Immunol (2021) 137:52–6. doi: 10.1016/j.molimm.2021.06.013 34214829

[21] Okazaki T Maeda A Nishimura H Kurosaki T Honjo T. PD-1 immunoreceptor inhibits b cell receptor-mediated signaling by recruiting src homology 2-domain-containing tyrosine phosphatase 2 to phosphotyrosine. Proc Natl Acad Sci USA (2001) 98:13866–71. doi: 10.1073/pnas.231486598 PMC61133 11698646

[22] Iwai Y Ishida M Tanaka Y Okazaki T Honjo T Minato N. Involvement of PD-L1 on tumor cells in the escape from host immune system and tumor immunotherapy by PD-L1 blockade. Proc Natl Acad Sci U.S.A. (2002) 99:12293–7. doi: 10.1073/pnas.192461099 PMC129438 12218188

[23] Petitprez F Meylan M de Reyniès A Sautès-Fridman C Fridman WH. The tumor microenvironment in the response to immune checkpoint blockade therapies. Front Immunol (2020) 11:784. doi: 10.3389/fimmu.2020.00784 32457745 PMC7221158

[24] Pagès F Galon J Dieu-Nosjean MC Tartour E Sautès-Fridman C Fridman WH. Immune infiltration in human tumors: a prognostic factor that should not be ignored. Oncogene (2010) 29:1093–102. doi: 10.1038/onc.2009.416 19946335

[25] Vinayak S Gray RJ Adams S Jensen KC Manola J Goldstein AAJ. Association of increased tumor-infiltrating lymphocytes (TILs) with immunomodulatory (IM) triple-negative breast cancer (TNBC) subtype and response to neoadjuvant platinum-based therapy in PrECOG0105. J Clin Oncol (2014) 32(15_suppl):1000. doi: 10.1200/jco.2014.32.15_suppl.1000

[26] Catacchio I Silvestris N Scarpi E Schirosi L Scattone A Mangia A. Intratumoral, rather than stromal, CD8+ T cells could be a potential negative prognostic marker in invasive breast cancer patients. Transl Oncol (2019) 12:585–95. doi: 10.1016/j.tranon.2018.12.005 PMC6350084 30682679

[27] Salgado R Denkert C Demaria S Sirtaine N Klauschen F Pruneri G.. The evaluation of tumor-infiltrating lymphocytes (TILs) in breast cancer: recommendations by an international tils working group 2014. Ann Oncol (2015) 26:259–71. doi: 10.1093/annonc/mdu450 PMC6267863 25214542

[28] Gong Y Liu YR Ji P Hu X Shao ZM. Impact of molecular subtypes on metastatic breast cancer patients: a SEER population-based study. Sci Rep (2017) 7:45411. doi: 10.1038/srep45411 28345619 PMC5366953

[29] Gaggianesi M Di Franco S Pantina VD Porcelli G D'Accardo C Verona F. Messing up the cancer stem cell chemoresistance mechanisms supported by tumor microenvironment. Front Oncol (2021) 11:702642. doi: 10.3389/fonc.2021.702642 34354950 PMC8330815

[30] Carvajal-Hausdorf D Altan M Velcheti V Gettinger SN Herbst RS Rimm DL. Expression and clinical significance of PD-L1, B7-H3, B7-H4 and TILs in human small cell lung cancer (SCLC). J Immunother Cancer (2019) 7:65. doi: 10.1186/s40425-019-0540-1 30850021 PMC6408760

[31] Mezheyeuski A Bergsland CH Backman M Djureinovic D Sjöblom T Bruun J. Multispectral imaging for quantitative and compartment-specific immune infiltrates reveals distinct immune profiles that classify lung cancer patients. J Pathol (2018) 244:421–31. doi: 10.1002/path.5026 29282718

[32] Halse H Colebatch AJ Petrone P Henderson MA Mills JK Snow H. Multiplex immunohistochemistry accurately defines the immune context of metastatic melanoma. Sci Rep (2018) 8:11158. doi: 10.1038/s41598-018-28944-3 30042403 PMC6057961

[33] Gorris MAJ Halilovic A Rabold K Duffelen A Wickramasinghe IN Verweij D. Eight-color multiplex immunohistochemistry for simultaneous detection of multiple immune checkpoint molecules within the tumor microenvironment. J Immunol (2018) 200:347–54. doi: 10.4049/jimmunol.1701262 29141863

[34] Marcelis L Antoranz A Delsupehe AM Biesemans P Ferreiro JF Debackere K. In-depth characterization of the tumor microenvironment in central nervous system lymphoma reveals implications for immune-checkpoint therapy. Cancer Immunol Immunother (2020) 69:1751–66. doi: 10.1007/s00262-020-02575-y PMC11027603 32335702

[35] Dieci MV Criscitiello C Goubar A Viale G Conte P Guarneri V. Prognostic value of tumor-infiltrating lymphocytes on residual disease after primary chemotherapy for triple-negative breast cancer: a retrospective multicenter study. Ann Oncol (2014) 25:611–8. doi: 10.1093/annonc/mdt556 PMC3933248 24401929

[36] Disis ML Stanton SE. Triple-negative breast cancer: immune modulation as the new treatment paradigm. Am Soc Clin Oncol Educ Book (2015) 35:e25–30. doi: 10.14694/EdBook_AM.2015.35.e25 25993181

[37] Luen SJ Salgado R Fox S Savas P Wong JE Clark E. Tumour-infiltrating lymphocytes in advanced HER2-positive breast cancer treated with pertuzumab or placebo in addition to trastuzumab and docetaxel: a retrospective analysis of the Cleopatra study. Lancet Oncol (2017) 18:52–62. doi: 10.1016/S1470-2045(16)30631-3 27964843 PMC5477653

[38] Loi S Sirtaine N Piette F Salgado R Viale G Rouas FV. Prognostic and predictive value of tumor-infiltrating lymphocytes in a phase III randomized adjuvant breast cancer trial in node-positive breast cancer comparing the addition of docetaxel to doxorubicin with doxorubicin-based chemotherapy: BIG 02–98. J Clin Oncol (2013) 31:860–7. doi: 10.1200/JCO.2011.41.0902 23341518

[39] Swisher SK Wu Y Castaneda CA Lyons GR Yang F Tapia C. Interobserver agreement between pathologists assessing tumor-infiltrating lymphocytes (TILs) in breast cancer using methodology proposed by the international tils working group. Ann Surg Oncol (2016) 23:2242–8. doi: 10.1245/s10434-016-5173-8 26965699

[40] Wang K Shen T Siegal GP Wei S. The CD4/CD8 ratio of tumor-infiltrating lymphocytes at the tumor-host interface has prognostic value in triple-negative breast cancer. Hum Pathol (2017) 69:110–7. doi: 10.1016/j.humpath.2017.09.012 28993275

[41] Khoury T Nagrale V Opyrchal M Peng X Wang D Yao S. Prognostic significance of stromal versus intratumoral infiltrating lymphocytes in different subtypes of breast cancer treated with cytotoxic neoadjuvant chemotherapy. Appl Immunohistochem Mol Morphol (2018) 26:523–32. doi: 10.1097/PAI.0000000000000466 PMC5550367 28187033

[42] Vasaturo A Galon J. Multiplexed immunohistochemistry for immune cell phenotyping, quantification and spatial distribution in situ. Methods Enzymol (2020) 635:51–66. doi: 10.1016/bs.mie.2019.10.002 32122553

[43] De Smet F Antoranz Martinez A Bosisio FM. Next-generation pathology by multiplexed immunohistochemistry. Trends Biochem Sci (2021) 46:80–2. doi: 10.1016/j.tibs.2020.09.009 33097382

[44] Shakya R Nguyen TH Waterhouse N Khanna R. Immune contexture analysis in immuno-oncology: applications and challenges of multiplex fluorescent immunohistochemistry. Clin Transl Immunol (2020) 9:e1183. doi: 10.1002/cti2.1183 PMC7541822 33072322

[45] Ouled Dhaou M Kossai M Morel AP Shisheboran MD Puisieux A Llorca FP. Zeb1 expression by tumor or stromal cells is associated with spatial distribution patterns of CD8+ tumor-infiltrating lymphocytes: a hypothesis-generating study on 113 triple negative breast cancers. Am J Cancer Res (2020) 10:3370–81. PMC7642672 33163276

[46] Mani NL Schalper KA Hatzis C Saglam O Tavassoli R Butler M. Quantitative assessment of the spatial heterogeneity of tumor-infiltrating lymphocytes in breast cancer. Breast Cancer Res (2016) 18:78. doi: 10.1186/s13058-016-0737-x 27473061 PMC4966732

[47] Ma J Zheng B Goswami S Meng L Zhang D Cao C. PD1 Hi CD8+ T cells correlate with exhausted signature and poor clinical outcome in hepatocellular carcinoma. J Immunother Cancer (2019) 7:331. doi: 10.1186/s40425-019-0814-7 31783783 PMC6884778

[48] Park MH Kwon SY Choi JE Gong G Bae YK. Intratumoral CD103-positive tumour-infiltrating lymphocytes are associated with favourable prognosis in patients with triple-negative breast cancer. Histopathology (2020) 77:560–9. doi: 10.1111/his.14126 32333690

[49] Yoo SY Park HE Kim JH Wen X Jeong S Cho N-Y. Whole-slide image analysis reveals quantitative landscape of tumor-immune microenvironment in colorectal cancers. Clin Cancer Res (2020) 26:870–81. doi: 10.1158/1078-0432.CCR-19-1159 31757879

[50] Noy R Pollard JW. Tumor-associated macrophages: from mechanisms to therapy. Immunity (2014) 41:49–61. doi: 10.1016/J.IMMUNI.2014.06.010 25035953 PMC4137410

[51] Kim YJ Won CH Lee MW Choi JH Chang SE Lee WJ. Correlation between tumor-associated macrophage and immune checkpoint molecule expression and its prognostic significance in cutaneous melanoma. J Clin Med (2020) 9:E2500. doi: 10.3390/jcm9082500 32756500 PMC7465191

[52] Cersosimo F Lonardi S Bernardini G Telfer B Mandelli GE Santucci A. Tumor-associated macrophages in osteosarcoma: from mechanisms to therapy. Int J Mol Sci (2020) 21:E5207. doi: 10.3390/ijms21155207 32717819 PMC7432207

[53] Savas P Salgado R Denkert C Sotiriou C Darcy PK Smyth MJ. Clinical relevance of host immunity in breast cancer: from TILs to the clinic. Nat Rev Clin Oncol (2016) 13:228–41. doi: 10.1038/nrclinonc.2015.215 26667975

