# Supplementary material for: Quantitative high-throughput analysis of tumor infiltrating lymphocytes in breast cancer
Source: Front Oncol. 2022 Sep 5;12:901591. doi: 10.3389/fonc.2022.901591 (PMC9484474; doi:10.3389/fonc.2022.901591)
Supplement: Supplementary file 1 [file DataSheet_1.docx]

Supplementary Material


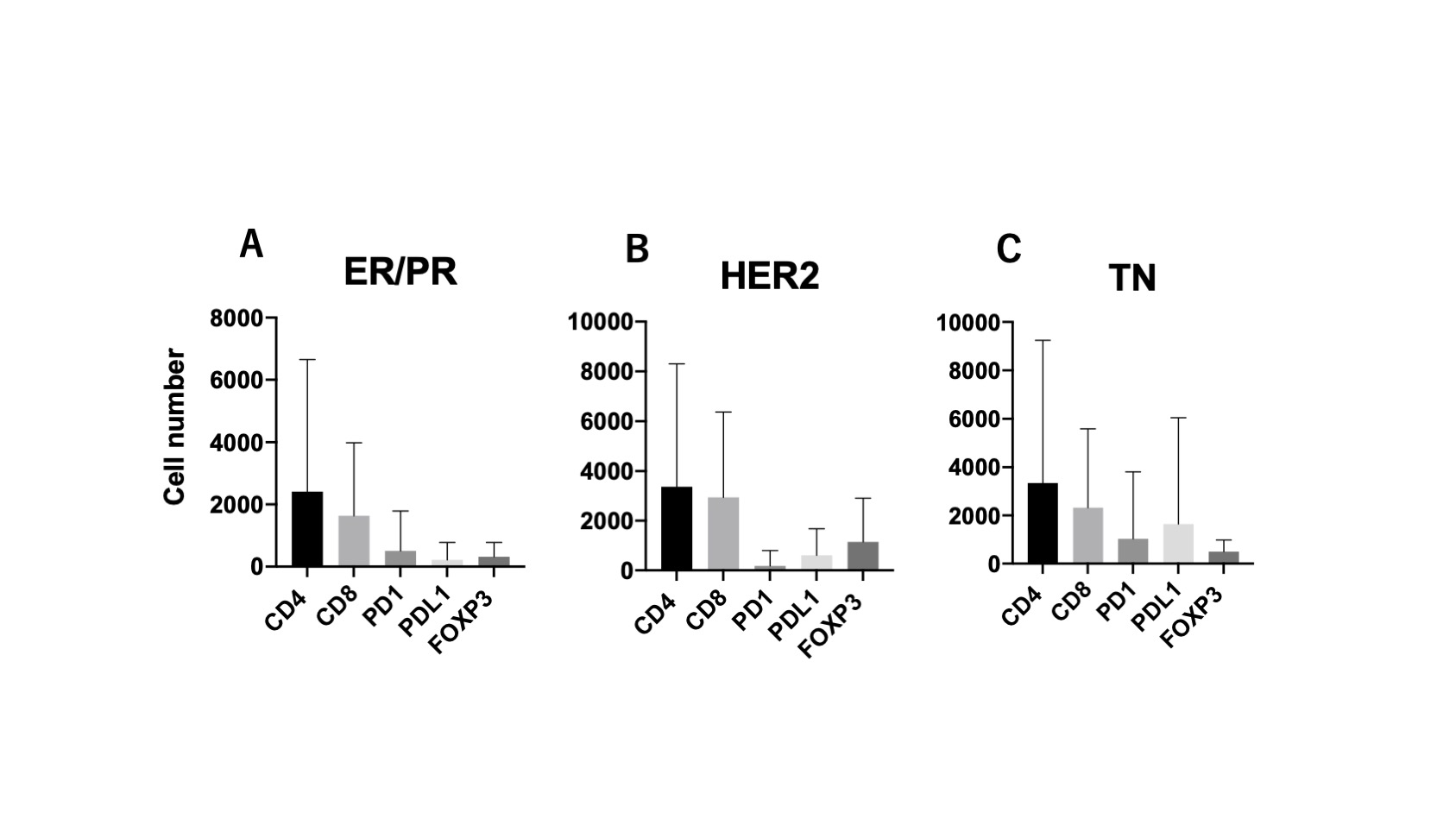


**Supplementary Figure 1.** Number of positive cells for stromal TIL (sTIL) markers stratified by (A) ER / PR, (B) HER2, and (C) TN subtypes. Average number of positive cells for sTIL markers (CD4, CD8, PD1, PDL1, and FOXP3) per 1 mm^2^ at 5 locations in each subtype. Error bars represent standard deviation.

**Supplementary Figure 2.** Comparison of stromal TIL (sTIL) markers among ER / PR, HER2, and TN subtypes. Average number of (A) CD4, (B) CD8, (C) PD1, (D) PDL1, and (E) FOXP3 positive sTILs among each subtype. *^,^ **, ***, and **** indicate p < 0.05, p < 0.01, p < 0.001 and p <0.0001, respectively (One-way ANOVA and Tukey’s multiple comparison test). Error bars represent standard deviation.


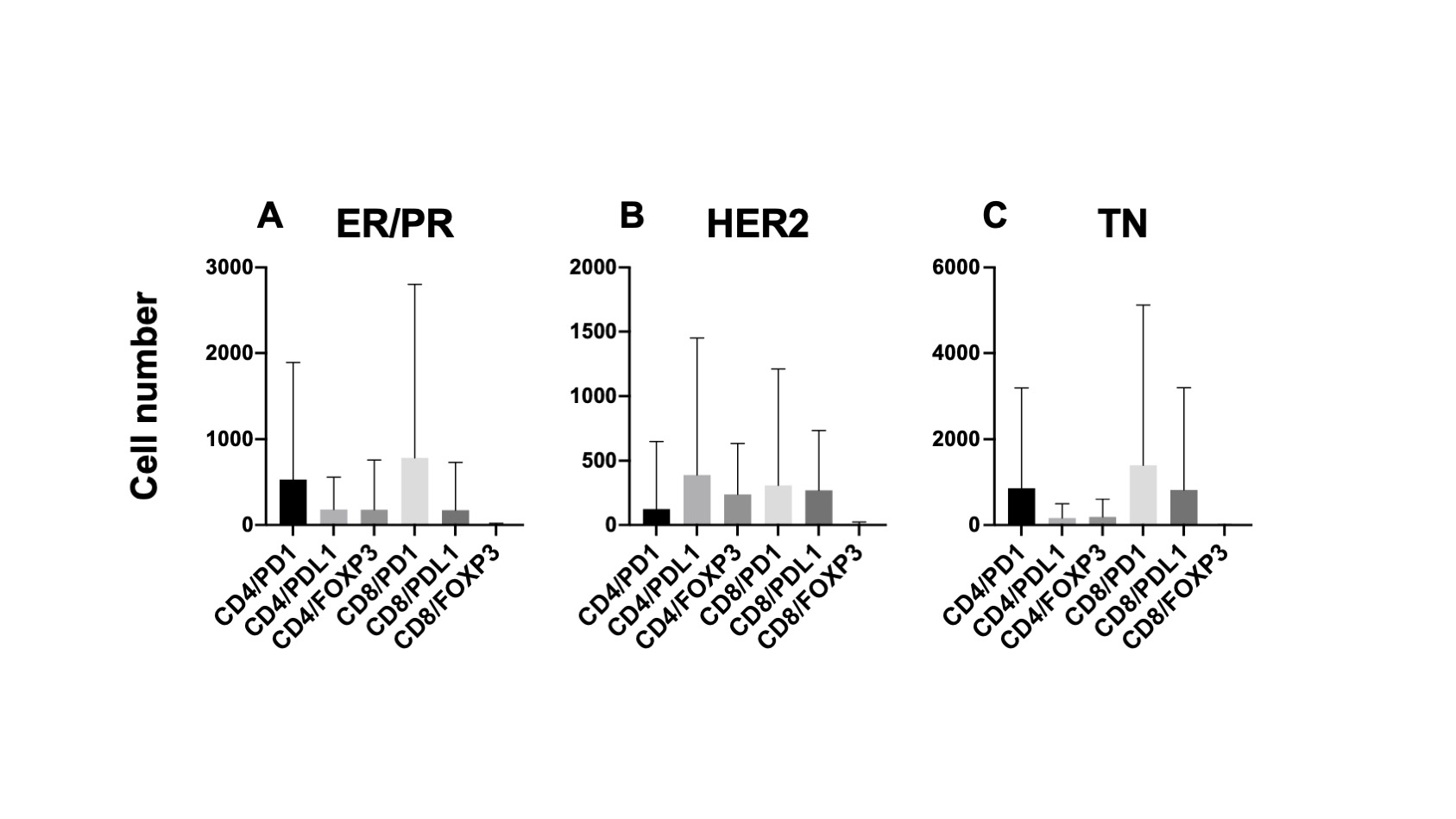


**Supplementary Figure 3.** Number of double-positive cells for stromal TIL (sTIL) markers stratified by (A) ER/PR, (B) HER2, and (C) TN subtypes. Average number of double-positive sTIL markers (CD4/PD1, CD4/PDL1, CD4/FOXP3, CD8/PD1, CD8/PDL1, and CD8/FOXP3) per 1 mm^2^ at 5 locations in each subtype. Error bars represent standard deviation.

^
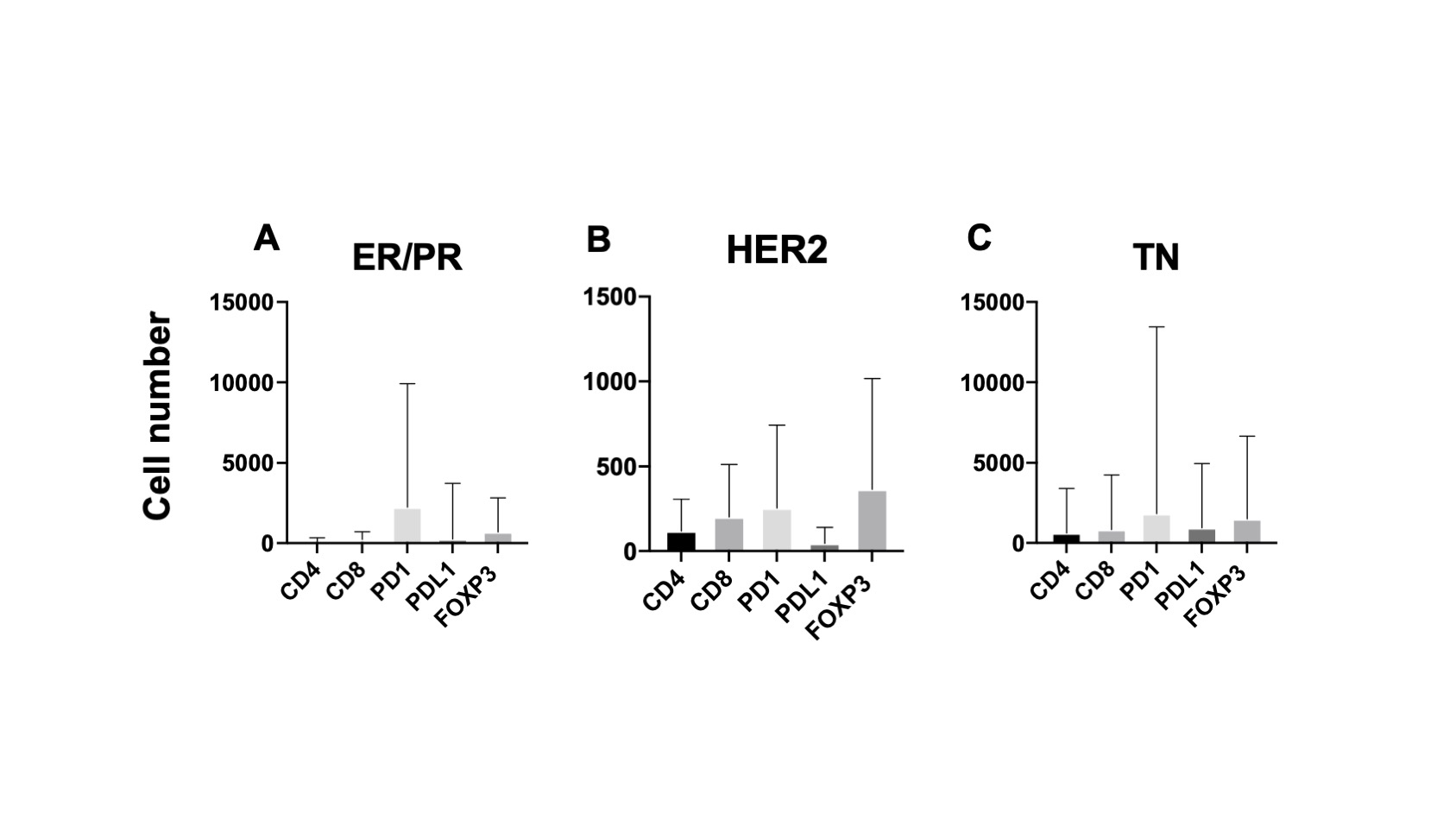
^

**Supplementary Figure 4.** Number of positive cells for intratumoral TIL (iTIL) markers stratified by (A) ER/PR, (B) HER2, and (C) TN subtypes. Average number of positive cells for iTIL marker (CD4, CD8, PD1, PDL1, and FOXP3) per 1 mm^2^ at 5 locations in each subtype. Error bars represent standard deviation.

^^

**Supplementary Figure 5.** Comparison of intratumoral TIL (iTIL) marker positive cell counts among ER / PR, HER2, and TN subtypes. Average number of (A) CD4, (B) CD8, (C) PD1, (D) PDL1, and (E) FOXP3 positive iTILs among each subtype. *^,^ ** indicate p < 0.05, and p < 0.01 respectively (One-way ANOVA and Tukey’s multiple comparison test). Error bars represent standard deviation.


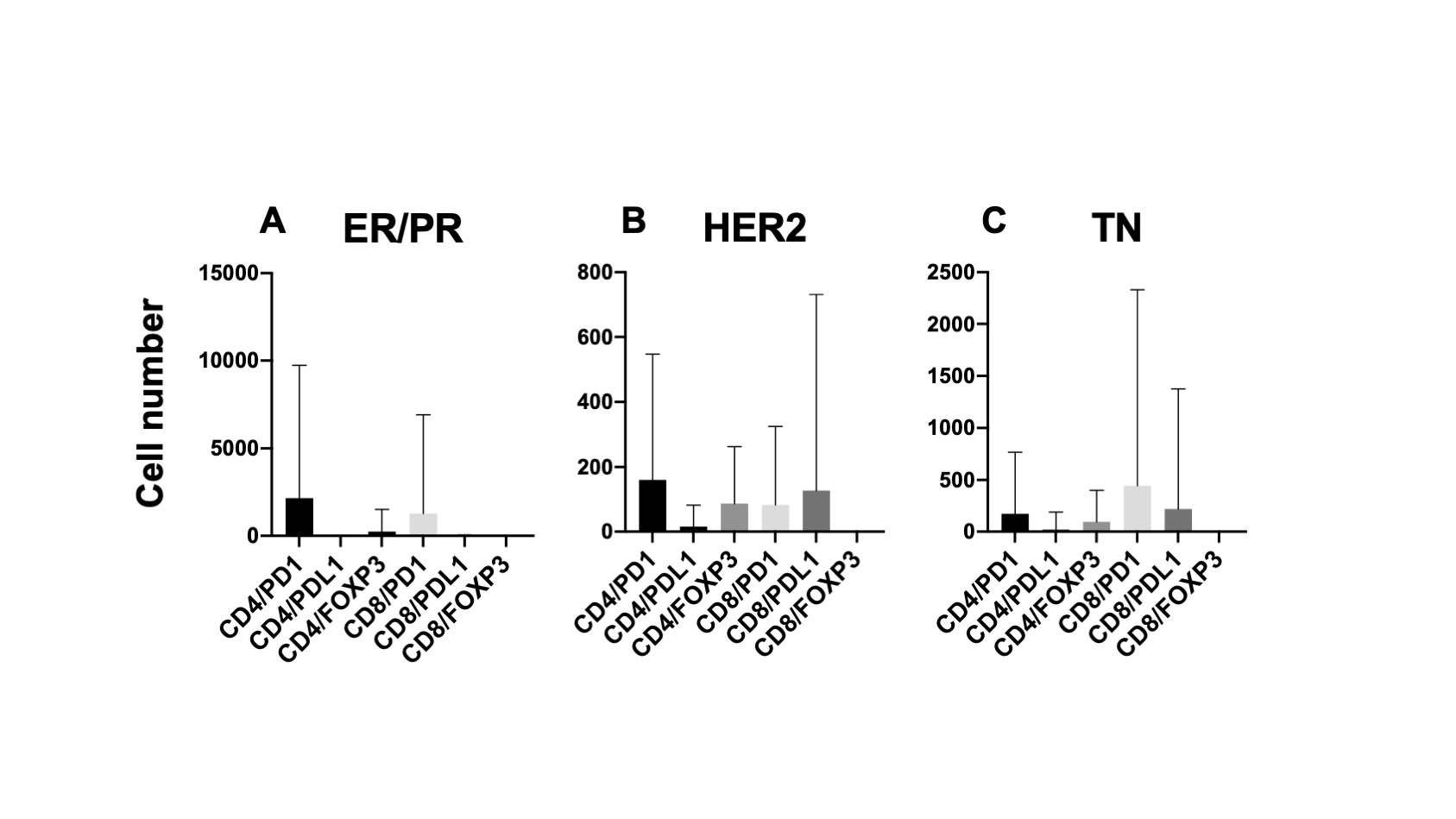


**Supplementary Figure 6.** Number of double-positive cells for intratumoral TIL (iTIL) markers stratified by (A) ER/PR, (B) HER2, and (C) TN subtypes. Average number of double-positive iTIL markers (CD4/PD1, CD4/PDL1, CD4/FOXP3, CD8/PD1, CD8/PDL1, and CD8/FOXP3) per 1 mm^2^ at 5 locations in each subtype. Error bars represent standard deviation.

^^

**Supplementary Figure 7.** Kaplan-Meier analysis for recurrence-free survival categorized by the amount of stromal TIL (sTIL) marker positive cells in triple-negative cases. Each sTIL marker was classified into “low” and “high” and analyzed as follows. (A) CD4-sTIL low; <1200 cells, high; >=1200 cells, (B) CD8-sTIL low; <1100 cells, high; >=1100 cells, (C) FOXP3-sTIL low; < 450 cells, high; >= 450 cells, (D) PD1-sTIL low; < 200 cells, high; >=200 cells, (E) PDL1-sTIL low; <200 cells, high; >=200 cells.

**Supplementary Figure 8.** Kaplan-Meier analysis for recurrence-free survival categorized by the amount of intratumoral TIL (iTIL) marker positive cells in triple-negative cases. Each sTIL marker was classified into “low” and “high” and analyzed as follows. (A) CD4-iTIL low; <115 cells, high; >=115 cells, (B) CD8-iTIL low; <120 cells, high; >=120 cells, (C) FOXP3-iTIL low; <300 cells, high; >=300 cells, (D) PDL1-iTIL low; <50 cells, high; >=50 cells, (E) PD1-iTIL low; <250 cells, high; >=250 cells,

**Supplementary Table 1.** Proportions of sTILs by single marker analysis and classified by breast cancer subtypes

| Marker | Subtype | Min(%) | Median(%) | Max(%) | SD |
| --- | --- | --- | --- | --- | --- |
| CD4 | ER/PR | 0.0 | 6.4 | 82.5 | 17.4 |
|  | HER2 | 0.0 | 7.4 | 62.0 | 14.1 |
|  | TN | 0.0 | 4.7 | 77.5 | 17.5 |
| CD8 | ER/PR | 0.0 | 5.9 | 44.4 | 10.1 |
|  | HER2 | 0.1 | 10.8 | 54.0 | 11.5 |
|  | TN | 0.0 | 6.6 | 44.9 | 10.7 |
| FOXP3 | ER/PR | 0.0 | 1.0 | 11.9 | 2.2 |
|  | HER2 | 0.0 | 3.4 | 25.3 | 4.3 |
|  | TN | 0.0 | 1.7 | 13.1 | 2.6 |
| PD1 | ER/PR | 0.0 | 0.6 | 96.3 | 8.7 |
|  | HER2 | 0.0 | 0.1 | 13.5 | 2.2 |
|  | TN | 0.0 | 0.6 | 43.0 | 8.4 |
| PDL1 | ER/PR | 0.0 | 0.2 | 15.2 | 2.4 |
|  | HER2 | 0.0 | 0.7 | 27.0 | 5.1 |
|  | TN | 0.0 | 1.1 | 57.5 | 11.5 |

**Supplementary Table 2.** Proportions of double-positive sTILs classified by breast cancer subtypes

| Marker | Subtype | Min(%) | Median(%) | Max(%) | SD |
| --- | --- | --- | --- | --- | --- |
| CD4/PD1 | ER/PR | 0.0 | 34.0 | 50.1 | 7.4 |
|  | HER2 | 0.0 | 8.0 | 14.4 | 1.9 |
|  | TN | 0.0 | 0.6 | 49.3 | 7.6 |
| CD4/PDL1 | ER/PR | 0.0 | 15.0 | 19.8 | 2.5 |
|  | HER2 | 0.0 | 3.5 | 36.4 | 6.7 |
|  | TN | 0.0 | 0.1 | 10.2 | 1.7 |
| CD4/FOXP3 | ER/PR | 0.0 | 0.2 | 17.0 | 0.0 |
|  | HER2 | 0.0 | 0.5 | 4.7 | 0.0 |
|  | TN | 0.0 | 0.2 | 7.6 | 0.0 |
| CD8/PD1 | ER/PR | 0.0 | 0.7 | 51.2 | 0.1 |
|  | HER2 | 0.0 | 0.2 | 22.4 | 0.0 |
|  | TN | 0.0 | 0.8 | 66.1 | 0.1 |
| CD8/PDL1 | ER/PR | 0.0 | 6.0 | 19.8 | 2.1 |
|  | HER2 | 0.0 | 3.2 | 9.0 | 1.5 |
|  | TN | 0.0 | 0.2 | 36.4 | 6.7 |
| CD8/FOXP3 | ER/PR | 0.0 | 0.0 | 0.5 | 0.0 |
|  | HER2 | 0.0 | 0.0 | 0.2 | 0.0 |
|  | TN | 0.0 | 0.0 | 0.0 | 0.0 |

**Supplementary Table 3.** Proportions of iTILs by single marker analysis and classified by breast cancer subtypes

| Marker | Subtype | Min(%) | Median(%) | Max(%) | SD |
| --- | --- | --- | --- | --- | --- |
| CD4 | ER/PR | 0.0 | 0.1 | 14.4 | 1.1 |
|  | HER2 | 0.0 | 0.1 | 1.9 | 0.4 |
|  | TN | 0.0 | 0.1 | 16.1 | 1.7 |
| CD8 | ER/PR | 0.0 | 0.2 | 5.6 | 0.8 |
|  | HER2 | 0.0 | 0.2 | 2.0 | 0.4 |
|  | TN | 0.0 | 0.3 | 8.0 | 1.2 |
| FOXP3 | ER/PR | 0.0 | 0.4 | 45.9 | 5.5 |
|  | HER2 | 0.0 | 0.5 | 3.3 | 0.6 |
|  | TN | 0.0 | 0.4 | 21.3 | 3.7 |
| PD1 | ER/PR | 0.0 | 0.5 | 96.9 | 15.5 |
|  | HER2 | 0.0 | 0.2 | 6.4 | 1.1 |
|  | TN | 0.0 | 0.3 | 34.9 | 4.8 |
| PDL1 | ER/PR | 0.0 | 0.0 | 4.2 | 8.2 |
|  | HER2 | 0.0 | 0.0 | 0.9 | 0.2 |
|  | TN | 0.0 | 0.1 | 22.8 | 3.4 |

**Supplementary Table 4.** Proportions of double-positive iTILs classified by breast cancer subtypes

| Marker | Subtype | Min(%) | Median(%) | Max(%) | SD |
| --- | --- | --- | --- | --- | --- |
| CD4/PD1 | ER/PR | 0.0 | 0.0 | 93.7 | 10.2 |
|  | HER2 | 0.0 | 0.0 | 5.9 | 0.9 |
|  | TN | 0.0 | 0.0 | 7.8 | 1.0 |
| CD4/PDL1 | ER/PR | 0.0 | 0.0 | 0.4 | 0.0 |
|  | HER2 | 0.0 | 0.0 | 0.3 | 0.1 |
|  | TN | 0.0 | 0.0 | 4.4 | 0.4 |
| CD4/FOXP3 | ER/PR | 0.0 | 0.0 | 17.9 | 1.9 |
|  | HER2 | 0.0 | 0.1 | 1.3 | 0.3 |
|  | TN | 0.0 | 0.0 | 3.3 | 0.5 |
| CD8/PD1 | ER/PR | 0.0 | 0.0 | 60.9 | 6.9 |
|  | HER2 | 0.0 | 0.0 | 2.2 | 0.3 |
|  | TN | 0.0 | 0.0 | 23.0 | 2.6 |
| CD8/PDL1 | ER/PR | 0.0 | 0.0 | 0.6 | 0.1 |
|  | HER2 | 0.0 | 0.0 | 0.9 | 0.2 |
|  | TN | 0.0 | 0.0 | 11.0 | 1.2 |
| CD8/FOXP3 | ER/PR | 0.0 | 0.0 | 0.0 | 0.0 |
|  | HER2 | 0.0 | 0.0 | 0.0 | 0.0 |
|  | TN | 0.0 | 0.0 | 0.0 | 0.0 |
